# Facile synthesis and thermoluminescence properties of nano bio-ceramic β-Ca_2_P_2_O_7_:Dy phosphor irradiated with 75 meV C^6+^ ion beam

**DOI:** 10.1038/s41598-020-78365-4

**Published:** 2020-12-03

**Authors:** Karan Kumar Gupta, S . J. Dhoble, Aleksander R. Krupski

**Affiliations:** 1 Department of Chemical Engineering, National Taiwan University, Taipei, Taiwan, ROC; 2 Department of Physics, RTM Nagpur University, Nagpur, 440033 India; 3 Department of Physics, University of Toronto, 60 St. George St., Toronto, ON M5S 1A7 Canada; 4 A A Krupski Ltd. Scientific Consulting, Hamilton, ON Canada

**Keywords:** Materials science, Condensed-matter physics

## Abstract

Dy^3+^ doped β-Ca_2_P_2_O_7_ phosphor has been synthesized using wet chemical method. The scanning electron microscopy (SEM) and transmission electron microscopy (TEM) analysis confirmed the formation of β-Ca_2_P_2_O_7_:Dy nano-phosphors. However, photoluminescence (PL) study was carried out to confirm the presence of dopant ion in the host matrix of β-Ca_2_P_2_O_7_:Dy material. Thermoluminescence (TL) glow curves of β-Ca_2_P_2_O_7_ were recorded for different concentrations of Dy^3+^ after exposure to various fluences of C^6+^ ion beam (75 meV). TL sensitivity of β-Ca_2_P_2_O_7_:Dy^3+^ (0.1 mol%) phosphor was 3.79 times more than commercially available CaSO_4_:Dy^3+^. TRIM code based on the Monte Carlo simulation was used to calculate the absorbed doses, ion range and main energy loss. Glow curve de-convolution (GCD) method was used to determine the number of TL peaks and their trapping parameters. The wide linear response of β-Ca_2_P_2_O_7_ nanoparticles along with high stability of TL glow curve makes this nanomaterial a good candidate for C^6+^ ion beam dosimetry.

## Introduction

TL is simple and popular techniques used for the dosimetry of ionizing radiations^1–3^. The amount of dose absorbed by the material is calculated on the basis of light emitted during TL measurements. The emitted light is directly proportional to amount of dose absorbed by the material. The increased value of dose enhances the TL output emission up to a certain limit of doses. Now a days this technique is widely studied to use in dosimetry of heavy charged particles (HCPs)^4,5^. TL materials display varying TL responses to high energy photons and HCP beams^6^. This might be due to the variations in spatial dose distribution of the radiation. Dosimetry of heavy ion beams find importance in diagnostic and therapeutic applications^7^. The existence of Bragg peak region and greater relative biological effectiveness (RBE) due to high linear energy transfer (LET) of carbon ion beam makes it an important tool in cancer/ tumor therapy. The RBE value for carbon ion beam increases up to a particular value of LET while the RBE value of photon and proton beams does not change significantly as the LET increases^7^. The major advantage of carbon ion beam is maximum dose deposition at the Bragg peak region which is better than that of proton or photon beams^7^. Conventional radiation beams dissipates its energy throughout its path and thus provide normal tissue complication whereas HCPs deposits their maximum energy to a particular confined region of targeted volume with least scattering and negligible angular energy straggling effects.

Heavy ion irradiation is also a unique tool for modifying the optical and electronic properties of inorganic materials^8^. When HCPs passes through materials, it loses energy via intense electronic excitations resulting in non-equilibrium conditions which help the system in achieving unique properties^9^. In the case of insulator or semiconducting phosphors, this inhomogeneous energy deposition may lead to the production of new color centers, and/or atomic vacancies (point defects) that modify the luminescence properties of materials^9^. TL is a well-known and very sensitive method for the characterization of these defects in solids.

Rare earth activated phosphate phosphors are recently used in solid state lighting and dosimetry applications due to their wide band gap, color tunability, and high thermal stability^10–12^. Calcium pyrophosphate is one of the most superior ceramics used as biomaterial in biotechnological applications due to absence of toxicity in their constituents. Some studies on TL properties of Ca_2_P_2_O_7_ doped with Tb^3+^, Eu^3+^, Ce^3+^ and Tm^3+^ have been reported in the literature^13–16^. The TL glow curve of Ca_2_P_2_O_7_:RE is composed of three peaks in which first peak is below 100 °C, second peak is around 150 °C and third peak is above 200 °C^16^. In the past few years, TL investigations on phosphate based phosphors such as LiMgPO_4_:Tb, Li_4_P_2_O_7_:Cu, NaLi_2_PO_4_:Eu, Sr_5_(PO_4_)_3_F:Dy and Li_2_BaP_2_O_7_:Dy have been carried out by many investigators^12,17–20^. Their observation indicates the synthesized phosphors are suitable for ionizing radiation dosimetry because of their high sensitivity and high stability of TL signal. However, the macro particles show early saturation of TL response even at low fluences of ion beam^21,22^. However, the early saturation of TL response can be overcome by using very tiny particles such as nano-scale TLD materials^5^. In the present work, we study the TL response of nanocrystalline β-Ca_2_P_2_O_7_:Dy phosphor irradiated by 75 meV C^6+^ ion beam in the fluence range of 2 × 10^10^ ions/cm^2^ to 1 × 10^12^ ions/cm^2^. The ion induced TL glow curves were well studied for its trapping parameters and other ion beam parameters using GCD functions and Monte Carlo SRIM 2013 simulations, respectively.

## Experimental

### Synthesis technique

β-Ca_2−x_P_2_O_7_:_x_Dy^3+^ (x = 0.0005, 0.001, 0.003, and 0.005) nano phosphors were synthesized by using simple wet chemical method. The chemicals used were Ca(NO_3_)_2_, (NH_4_)_2_HPO_4_ (99.9% pure, Loba make) and Dy_2_O_3_ (99.99% pure, Himedia) as an initial precursors. Dysprosium in the form of oxide was converted to nitrate by dissolving Dy_2_O_3_ in dilute nitric acid. The stoichiometric amount of calcium nitrate was dissolved separately in double distilled deionized water through a continuous stirring on a hot plate. Dysprosium nitrate as a dopant and ethanol (150 mL) as a surfactant was added drop wise to the whole solution (mixed solution). The ethanol was used to prevent agglomeration and control the size of particles. Further, ammonium phosphate solution was transferred into a burette and mounted above the beaker (mixed solution). As the ammonium phosphate solution was added to the mixed solution, amorphous nanoparticles were formed. The surfactant added to the mixed solution modulates the available surface energy of the particles so that the surface tension decreases and allowing more particles to escape the aglomoration process results in lowering the mean particle size. Without extracting the precipitate from the mixed solution, the extra water of the solution was evaporated using a hot plate maintained at 40 °C. The extra water of the mixed solution has not been decanted, since the formation of more soluble calcium phosphate complexes, *i.e.* less precipitation of Ca-complex in reaction product is possible which may lead to the changes or appearance of other phases of alkaline phosphates in the final product. The amorphous sample was then transferred to silica crucible and dried at 60 to 80 °C for 12 h. Further annealing of sample was carried out at 600 °C to remove the presence of extra ammonia and water molecule. Another purpose of annealing at 600 °C is the conversion of amorphous Ca-Dy-P-O–H nanoparticles to crystalline β-Ca_2−x_P_2_O_7_:_x_Dy^3+^ nanoparticles.

### Characterizations techniques

The formation of compound and phase purity was confirmed by using powder X-ray diffraction technique. XRD pattern was observed at room temperature by using Bruker D8Advance diffractometer with Cu target (CuK_α_ line λ = 1.5406 Å). The scanning step was kept at 0.02° in 2θ range selected from 20° to 70°. Fourier transform infra-red (FTIR) spectroscopy studies have been performed on a Bruker alpha ATR set up within the scanning range of 600–4000 cm^−1^ with precision of 4 cm^−1^.To study the morphology of the synthesized sample it was coated with gold and then studied by field emission scanning electron microscopy (FE-SEM) [MIRA II LMH from TESCAN], operating at 25 kV. TEM analysis was carried out using Hitachi H-8100 (accelerating voltage up to 200 kV). Particle size analysis was carried out using laser diffraction spectroscopy (LS 230). We have mixed 50 mg of phosphor in 5 mL of water and sonicated for 30 min before performing particle size analysis experiment. Photoluminescence study was carried out using Shimadzu RF-5301 PC spectrophotoflurometer. The slit width during each PL measurements was kept at 1.5 nm. According to the previous work of Salah et al. samples in the form of pellets were irradiated at room temperature by 75 meV C^6+^ ion beam at different ion fluences in the range of 2 × 10^10^ to 1 × 10^12^ ions/cm^2^^5^. Harshaw TLD reader (3500HT) was used to record the TL glow curves. Each TL measurements used 5 mg samples and heating rate was kept at 5 °C/s.

## Results and discussion

### X-ray diffraction pattern

**Figure 1:**
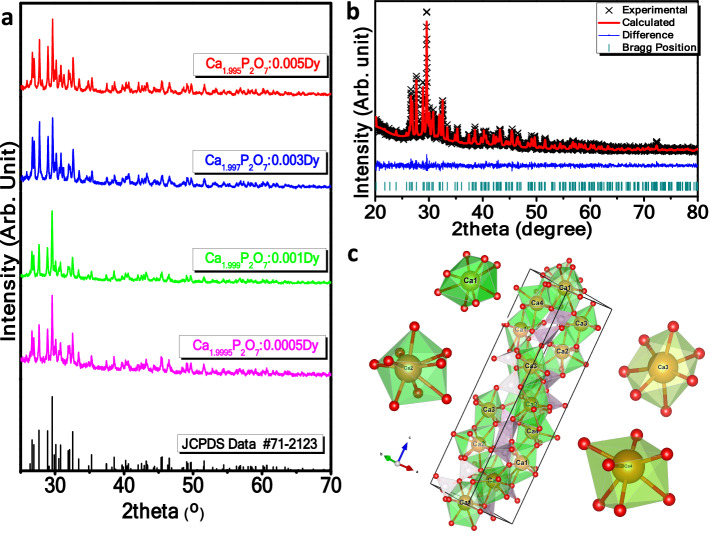
(**a**) XRD pattern of Dy^3+^ doped β-Ca_2_P_2_O_7_ phosphor, (**b**) Rietveld refined XRD pattern and (**c**) crystal structure of 0.001 mol Dy^3+^ doped β-Ca_2_P_2_O_7_ phosphor synthesized by wet chemical method was generated by visualization for electronic and structural analysis (VESTA, Ver. 3.2.1) program ( https://jp-minerals.org/vesta/en/download.html).

Figure 1 shows the XRD pattern of β-Ca_2_P_2_O_7_:Dy^3+^ samples. The sharp and intense peaks in the XRD patterns show the crystalline nature of the prepared material. The diffraction peaks can be indexed properly with standard JCPDS data no. #71–2123. The XRD pattern exhibit prominent diffraction peaks of tetragonal structure of β-Ca_2_P_2_O_7_ associated to a space group P4_1_ (76).

XRD pattern of 0.001 mol Dy^3+^ doped β-Ca_2_P_2_O_7_ were refined using Topaz software with Rietveld technique. Rietveld refined XRD pattern of Ca_2_P_2_O_7_:0.001Dy^3+^ with experimental, calculated and residue pattern is shown in Fig. 1b. Experimental data has good match with the simulated data with refinement parameters like R_wp_, R_exp_, R_p_ and χ^2^ of 9.63, 8.65, 7.39 and 1.11, respectively. The initial input parameters needed for refinement of XRD pattern were generated from the previous data of Boudin et al.^23^ Lattice parameters obtained from refined data are a = b = 6.605 Å, c = 23.849 Å and unit cell volume is 1040.373 Å^3^. The crystal structure was generated by visualization for electronic and structural analysis (VESTA, Ver. 3.2.1) program using Rietveld refined output.cif file^24^. Crystal structure shows the existence of four independent crystallographic sites for Ca^2+^ ion in the β-form of Ca_2_P_2_O_7_^25^. Among the four sites, two calcium atoms such as Ca3 and Ca4 are surrounded by seven oxygen atoms forming distorted pentagonal bi-pyramidal coordination. The remaining two calcium atoms like Ca1 and Ca2 are surrounded by eight oxygen and nine oxygen atoms forming bi-capped and tricapped trigonal prisms geometries, respectively. The local environment around calcium atom does not have inversion symmetry which increases the probabilities of electric dipole transition if lanthanide ions are incorporated at Ca^2+^ site in β form of Ca_2_P_2_O_7_. The ionic radii of Dy^3+^/Ca^2+^ in seven, eight and nine coordination are 0.97/1.06, 1.027/1.12, and 1.083/1.18, respectively. The ionic radii and valence state of Dy^3+^ ion is very close to those of Ca^2+^, thus it can be predicted that the Dy^3+^ ions would occupy the Ca^2+^ lattice sites in the host structure.

### FTIR spectral analysis

**Figure 2:**
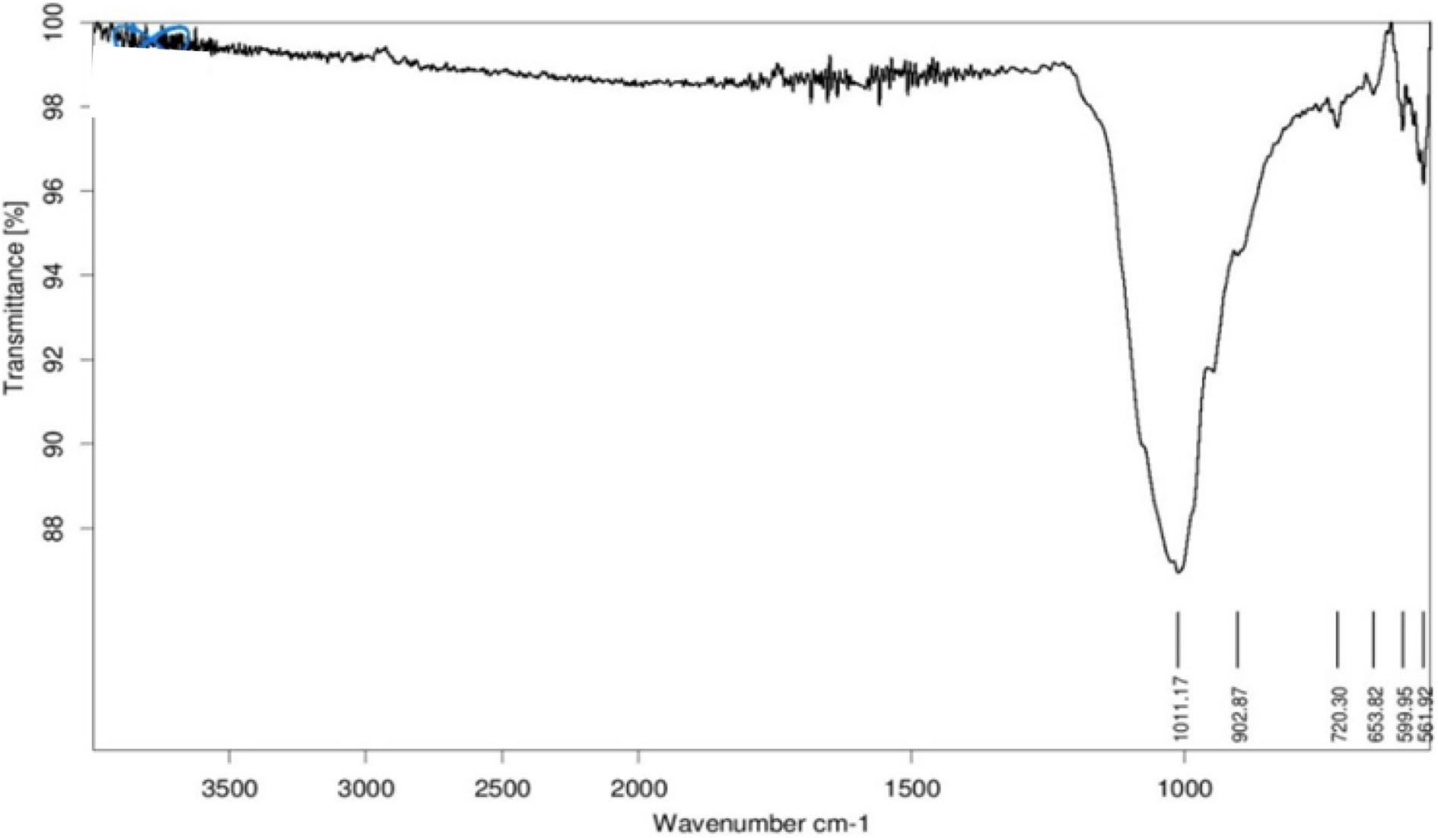
FTIR spectra of β-Ca_2_P_2_O_7_:Dy (0.1 mol%) synthesized via wet-chemical method.

Figure 2 represents the FTIR spectra of 0.1 mol% Dy^3+^ doped β-Ca_2_P_2_O_7_ phosphor. The presence of PO_4_^3−^, PO_3_^1−^ or P_2_O_7_^4−^ group can be confirmed with the help of IR spectra. Since the compound was synthesized by wet chemical method, presence of water molecule in the sample is possible through moisture absorbed from the atmosphere. The presence of water molecule can considerably affect the luminescence property of the phosphor under study. The FTIR spectra does not show any band related to IR absorption band of H_2_O molecule. The water molecules that are fused into the lattice structure of a crystalline compound yield specific sharp bands in the 1700–1600 cm^−1^ and 3800–3200 regions, due to O–H bending and stretching, respectively^26^. Since no any absorption peak was observed in this range this suggest the lack of H_2_O molecule in the sample. Two major ranges can be marked in the above spectra of the studied phosphate: a range (1100–900 cm^−1^) analogous to a symmetric and anti-symmetric stretching vibrations of the P-O-P bond present in diphosphate anion group as well as a range 650–550 cm^−1^ corresponding to the bending vibrations of O–P–O groups and lattice modes^27^. The peaks observed at 720 cm^−1^ is due to the symmetric stretching vibrations of the P-O-P bridges in P_2_O_7_^4−^ group^26^.

### SEM and TEM studies

**Figure 3:**
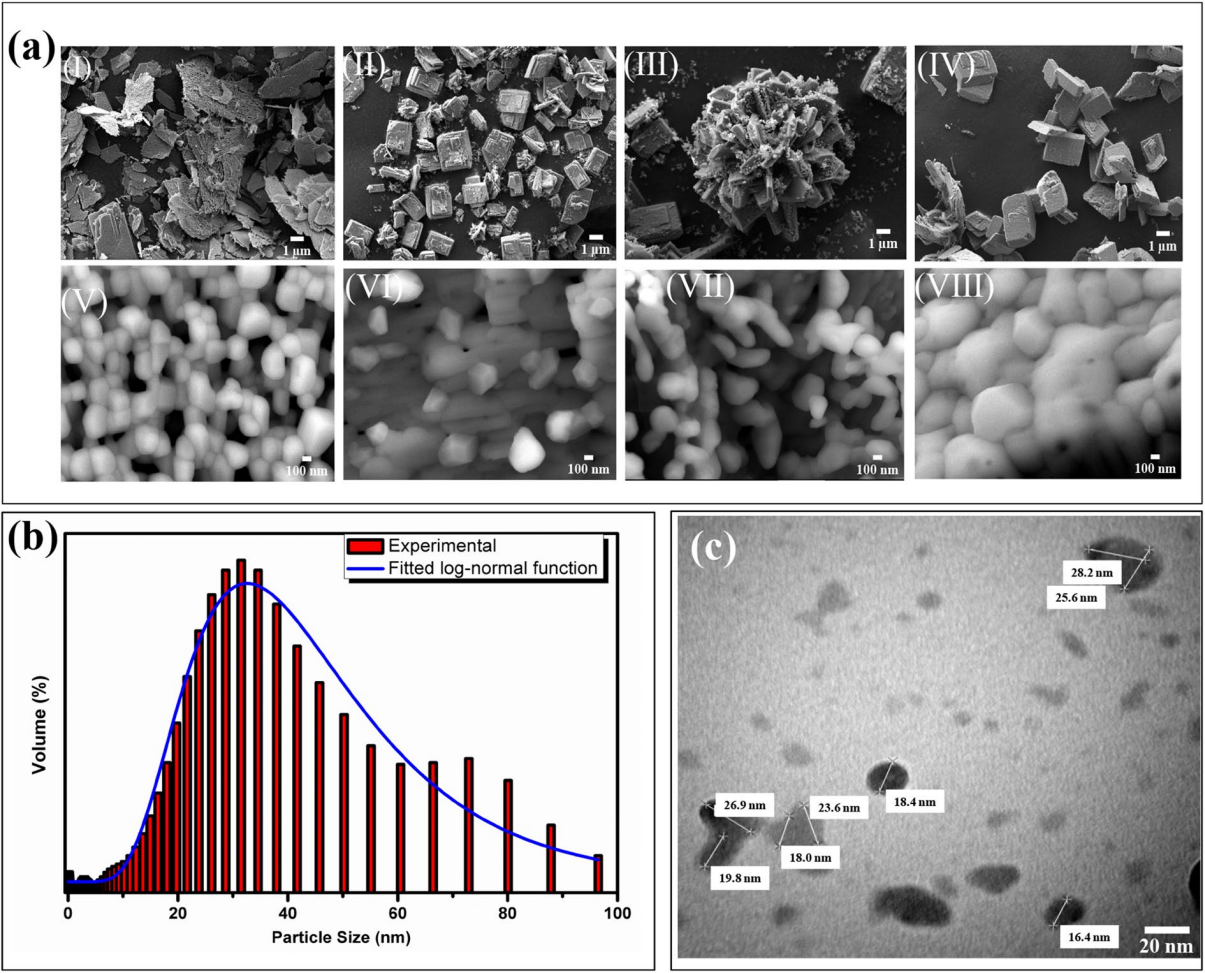
(**a**) SEM micrograph of (I, V) 0.0005 (II, VI) 0.001 (III, VII) 0.003 and (IV, VIII) 0.005 Dy^3+^ doped β-Ca_2_P_2_O_7_ phosphors at higher and lower magnifications (**b**) Measured size distribution of 0.001Dy^3+^ doped β-Ca_2_P_2_O_7_ phosphor (**c**) corresponding TEM image of 0.001Dy^3+^ doped β-Ca_2_P_2_O_7_ phosphor synthesized by wet chemical method and sintered at 600 °C.

The surface morphology and the crystallite size of the prepared phosphor were determined through SEM and TEM analysis. SEM image reveals that the particles have very less uniform shape and are highly agglomerated with broad size distribution. Thus, the correct estimation of particle size through SEM micrograph is not possible however a predication can be made. SEM images of each samples was recorded at two different magnifications and shown in Fig. 3a. The lower magnification of SEM images [Fig. 3a(I–IV)] shows the formation of thin and thick plate-like structure due to bulk agglomeration of tiny small nanoparticles of β-Ca_2_P_2_O_7_. Figure 3a(III) shows the flower-like structure made from the aggregation of thin and thick plate-like morphology while Fig. 3a(IV) shows the brick-like morphology made from dense agglomeration of small grains of β-Ca_2_P_2_O_7_. The SEM micrograph taken at higher magnification [Fig. 3a(V–VIII)] reveals the existence of various nanoparticles within the range of 18 to 30 nm showing the non-uniform morphology along with few spherical shaped grains. The characteristics like particle morphology, and crystallite size, may have impact towards the luminescence efficiency of the phosphor. The particle size in the range of nanometer show delayed saturation of TL response for a wide range of radiation doses. The average particle size estimated by particle size analyzer was found to be around 40 nm and shown in Fig. 3b.

In order to examine the nature of synthesized powder particles more precisely, TEM of β-Ca_2_P_2_O_7_:0.001Dy sample was carried out and shown in Fig. 3c. TEM image reveals highly agglomerated non-uniform nanoparticles having average grain size of less than 30 nm which is comparable with the crystallite size predicted by SEM data.

### Photoluminescence studies

**Figure 4:**
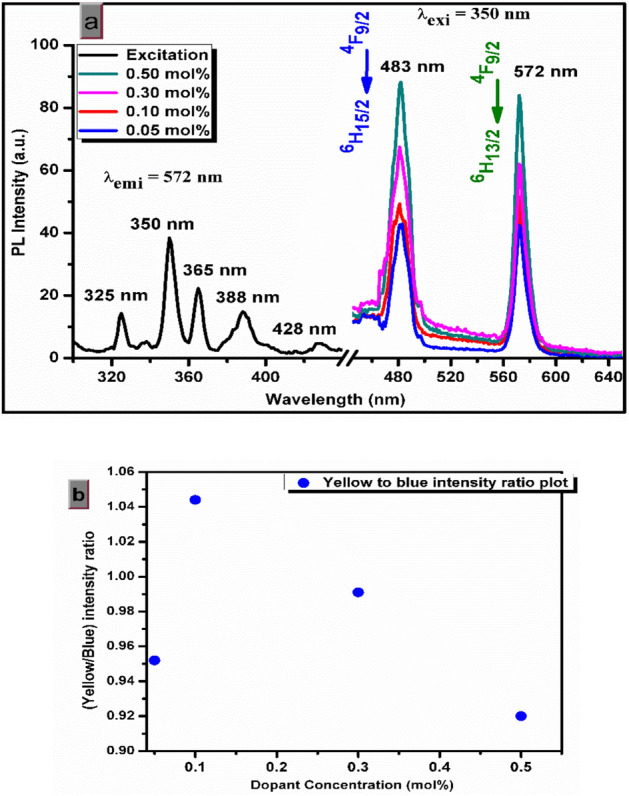
(**a**) Excitation and emission spectra of β-Ca_2_P_2_O_7_ phosphor (**b**) yellow to blue intensity ratio plot for different dopant concentration of Dy^3+^ ion.

The confirmation of presence and luminescence behavior of rare earth incorporation in the host lattice of β-Ca_2_P_2_O_7_ was investigated from PL emission and excitation spectra shown in Fig. 4. The addition of Dy^3+^ dopant inside β-Ca_2_P_2_O_7_ phosphor results in an intense white emission for an excitation of 350 nm while the un-doped samples does not show any PL emission hence, it has been not shown in emission spectra of Dy^3+^. Thus, we can easily say that the rare earth ions working as the luminescence centers and PL study confirms the presence of Dy^3+^ in the β-Ca_2_P_2_O_7_ host matrix.

The excitation spectra of β-Ca_2_P_2_O_7_:Dy is made up of a series of sharp bands in the region of 300–500 nm. The number of excitation peaks observed at 325, 350, 365, 388, and 428 are assigned to the ground state ^6^H_15/2_ to the excited state ^4^L_19/2_, ^6^P_7/2_, ^6^P_5/2_, ^4^I_13/2_, and ^4^G_11/2_, respectively^28^. The peaks located at 350, 365, and 388 are found to be more dominant than others. The emission spectra of β-Ca_(2−x)_P_2_O_7_:(x = 0.0005, 0.001, 0.003, 0.005)Dy^3+^ phosphors excited by 350 nm UV light reveals two dominating peaks at around (blue) 483 nm and (yellow) 572 nm, corresponding to ^4^F_9/2_ → ^6^H_15/2_ (electric dipole), ^4^F_9/2_ → ^6^H_13/2_ (magnetic dipole) transitions, respectively. The ^4^F_9/2_ → ^6^H_13/2_ transition belongs to the hypersensitive transition with ∆J = 2, which is strongly sensitive to change in environment of Dy^3+^^28^. The intensity ratio of hypersensitive transition to a non-hypersensitive transition of Dy^3+^ reveals the position of Dy^3+^ without any inversion center. The emission spectra for different molar concentration and Y/B (yellow to blue intensity) ratio is shown in Fig. 4a,b, respectively. The value of Y/B ratio for different concentration of Dy^3+^ is found to be changed from 0.952 to 0.920 suggests the substitution of Dy^3+^ ion at the site of divalent ion which results in the formation of defects and change in the local symmetry of Dy^3+^ with increasing concentration^29^.

### Thermoluminescence studies

**Figure 5:**
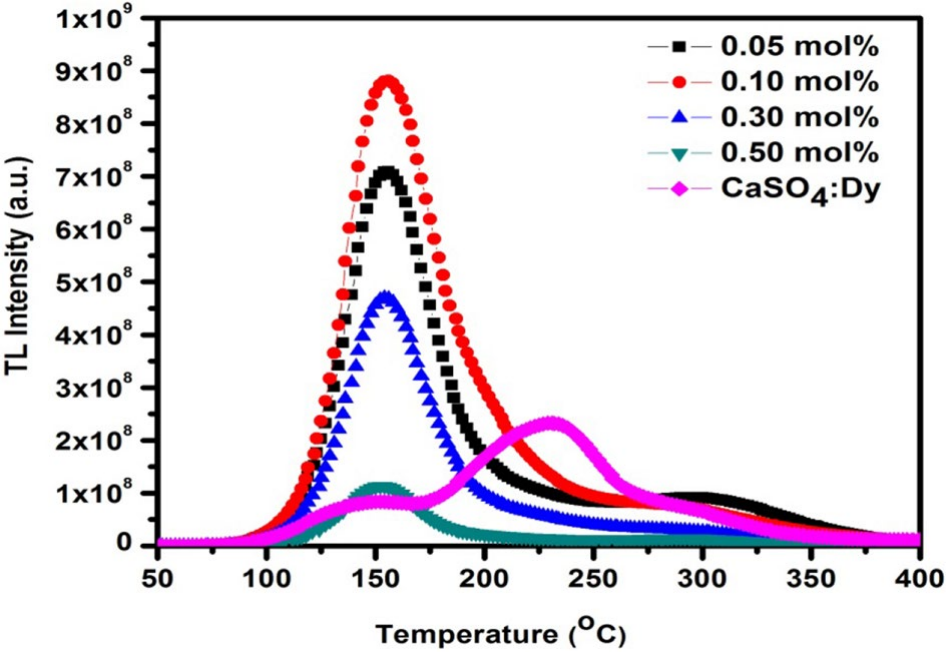
TL glow curves of C^6+^ ion irradiated β-Ca_2_P_2_O_7_:Dy^3+^ phosphor.

**Figure 6:**
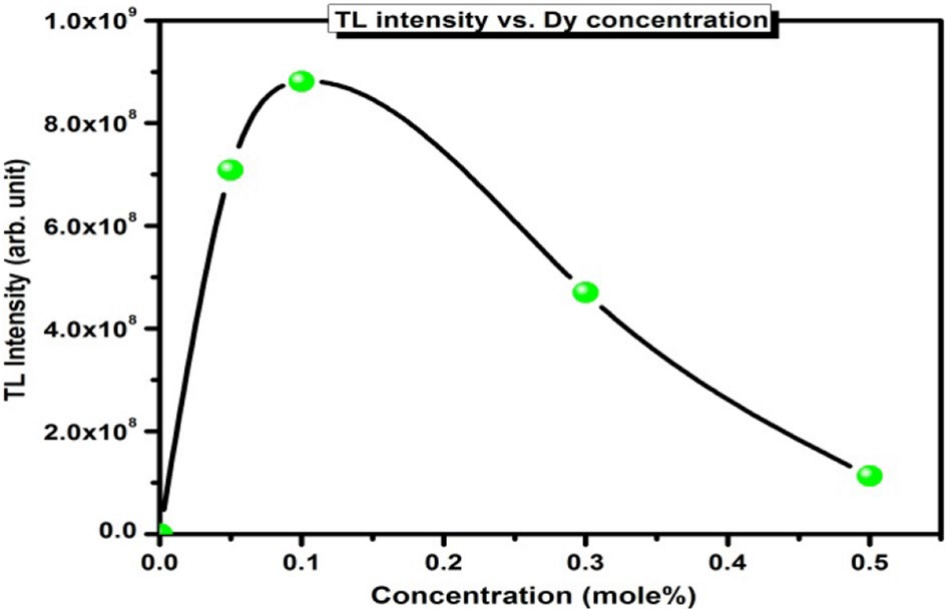
Variation of TL intensity as a function of Dy^3+^ ion concentrations.

The TL glow curves of β-Ca_2_P_2_O_7_ with different concentration of Dy^3+^ ions exposed to 1 × 10^11^ ions/cm^2^ fluence from a 75 meV C^6+^ ion beam is shown in Fig. 5. The figure shows that the glows curve structure of all the samples are almost identical for different concentrations of Dy^3+^ ions. The β-Ca_2_P_2_O_7_ samples exhibit simple glow curve peaking at 155 °C and a shouldered peak towards higher temperature side. The observed glow curve has the similar trend with the previous reported studies of Lopez et al.^16^. The nature of TL glow curve clearly indicates the presence of various types of defect centers and more than one overlapping traps. These traps releases charge carriers on thermal stimulation and finally recombine with their counterpart and give rise to diverse glow peaks with different heights. The TL response is highly sensitive to the quantity of doped impurity ion. So, the optimization of dopant (impurity) content is essential when TL sensitivity was taken into consideration. The variation in TL response has been observed with different concentrations of Dy^3+^ ions in β-Ca_2_P_2_O_7_ phosphor. This could be due to alteration in the number of optically active luminescent centers in β-Ca_2_P_2_O_7_ host. It can be seen from Fig. 6 that maximum TL intensity is observed for the 0.001 mol Dy^3+^ ion content and a further increase in Dy^3+^ ion concentration results in a decrease in TL intensity. The TL intensity found to quench just after 0.001 mol Dy^3+^ ion, might be due to the fact that more luminescence centres are generated initially and afterward with the increasing content of Dy^3+^ ions the distance between luminescence centres decreases leading to enhanced step by step interaction between luminescence centres at the higher concentration level of Dy^3+^ dopants, which result in decreased TL intensity^30,31^. TL of the CaSO_4_:Dy^3+^ phosphor has also been recorded to compare the TL sensitivity of β-Ca_2_P_2_O_7_ with standard commercial phosphor. It was observed that the sensitivity of the β-Ca_2_P_2_O_7_:(0.001)Dy phosphor is approximately 3.79 times more compared to the sensitivity of CaSO_4_:Dy^3+^.

### TL response curve

**Figure 7:**
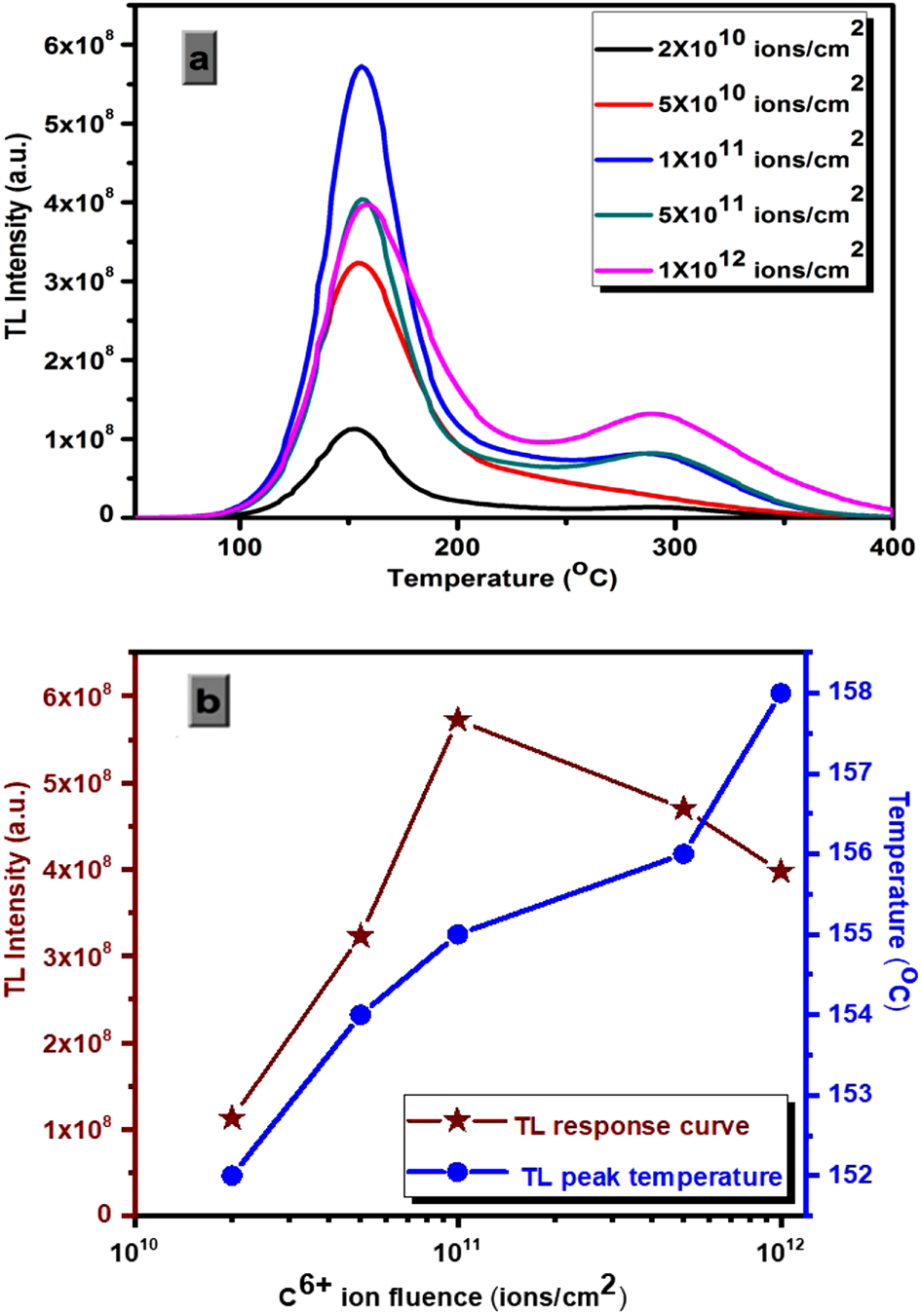
(**a**) TL glow curves and (**b**) TL response curve with peak temperature variation curve of nanocrystalline β-Ca_2_P_2_O_7_:Dy phosphor irradiated by the 75 meV C^6+^ ion beam at different fluences.

**Figure 8:**
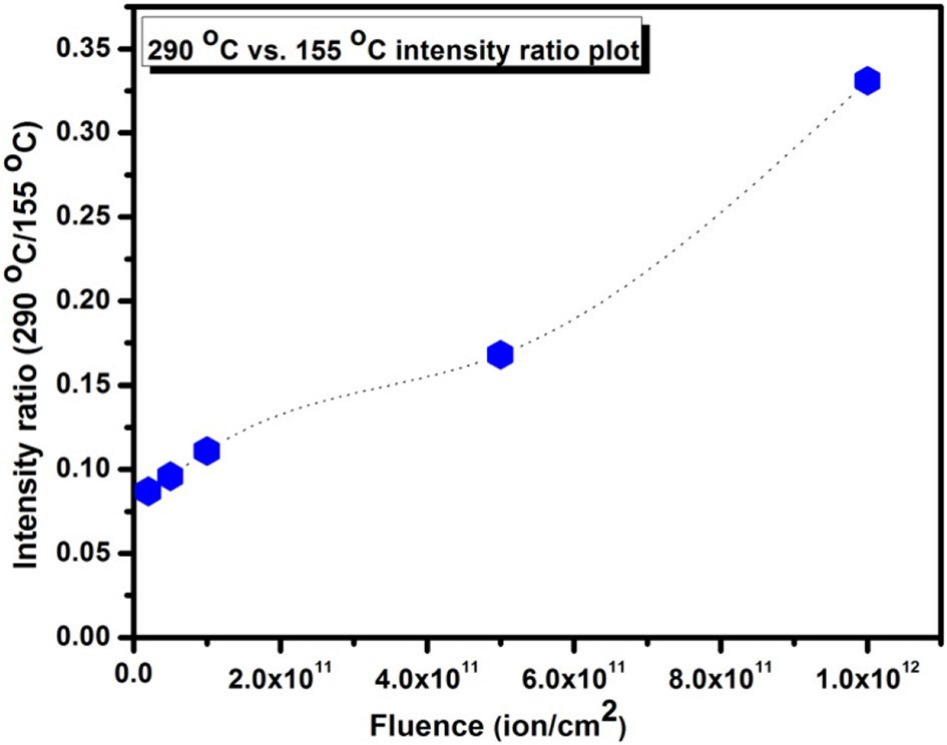
Intensity ratio of 290 to 155 °C peaks of β-Ca_2_P_2_O_7_:Dy nanocrystalline sample as a function of 75 meV C^6+^ ion beam fluence.

The TL glow curve of C^6+^ ion beam irradiated β-Ca_2_P_2_O_7_:(0.001)Dy phosphor in the fluence range of 2 × 10^10^ to 1 × 10^12^ ions/cm^2^ is shown in Fig. 7a. It is observed that the shape of TL glow curve does not change with the varying fluences, only a variation in sensitivity is observed. The intensity of prominent glow peak was considered for TL response analysis. The TL response curve as shown in Fig. 7 increases linearly with C^6+^ ion beam fluence up to 1 × 10^11^ ions cm^−2^ and thereafter it starts decreasing. A wide linear TL response has been observed for nanocrystalline β-Ca_2_P_2_O_7_:Dy material while other microcrystalline materials shows early saturation towards ion beam irradiation. The reason behind wide TL response of nanocrystalline phosphor is already explained by several authors in the earlier work^4,5^. A shifting of position of maximum glow peak temperature is observed (Fig. 7b) as the C^6+^ ion fluence rate has been increased from 2 × 10^10^ to 1 × 10^12^ ions/cm^2^, which suggests that the trap levels sparsely vary with change in ion fluence. This point clearly indicates the non-first order kinetic behavior of prominent glow peak. The intensity of higher temperature peaks increases more as the fluence increases above 1 × 10^11^ ions/cm^2^ while the intensity of prominent peak start decreasing above this particular ion fluence. The intensity ratio between the 290 and 155 °C peaks of nanocrystalline β-Ca_2_P_2_O_7_:Dy is plotted as a function of the ion beam fluence and is shown in Fig. 8. As seen in this figure, there is a slight change in the value of this ratio at low fluences while it drastically increases at higher fluences. The rapid growth in the intensity of the higher temperature peaks is also reported in several earlier studies on different TL materials irradiated by different ions^32^. The variation of these peaks is due to the changes in the population of the luminescent and trapping centres after highly increased value of energetic C^6+^ ions. Linearity and sub-linearity in TL sensitivity of β-Ca_2_P_2_O_7_:Dy can be explained in the framework of track interaction model (TIM)^33^. According to this model, exposure to HCP ion beam creates electron and hole pairs surrounding the ion track. Some of these charges are trapped near the track. The centers thus produced along these tracks are trapping centers (TCs) and luminescence centers (LCs). At low ion fluences, the TL signal arises during readout are only from the recombination of TCs and LCs occurs entirely within the same tracks. Since the distances between two neighboring tracks are large, charge carriers escaping the parent tracks are prevented by the competing non radiative centers or inactive trapping centers in inter track region and do not yield extra TL. Hence, the TL response is linear in the low fluence region. For higher fluences, the inter track regions are diminished and adjacent tracks begins to merge and overlap each other. The overlapping tracks reduce the effective ionization rate resulting in less number of trapped charge carriers. The full occupied TCs and LCs do not give extra TL, resulting sub-linearity and finally saturation. The reduced TL intensity at higher fluence is also caused by the induced stress/strain in the host lattice because of dense ionization. Such type of dense ionization produces clustering of defects that lead to the creation of voids and diffusion of defect center into un-treated part of the TL material^34^. Thus, the imperfection and stress along the ion track influences the recombination of charges and alters the shape, positions and intensities of the TL glow curve. However, the process becomes complicated when energetic ions get implanted inside the host lattice, and might have generated new kinds of defects.

### TL signal fading

**Figure 9:**
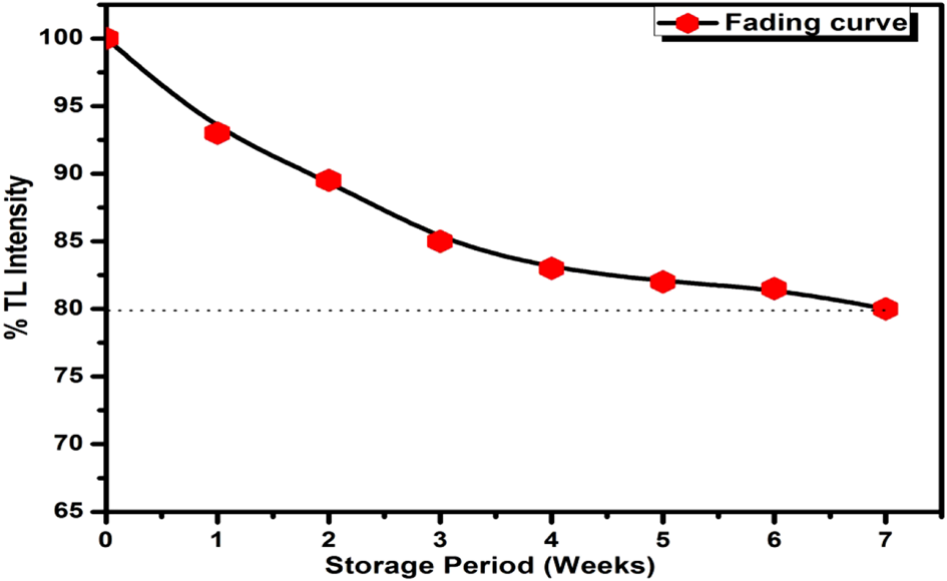
TL signal fading curve of β-Ca_2_P_2_O_7_:Dy nanocrystalline phosphor exposed to 1 × 10^11^ ions/cm^2^ of 75 meV C^6+^ ion beam over seven weeks of storage period.

The fading of TL signal of β-Ca_2_P_2_O_7_:(0.001)Dy phosphor irradiated with carbon ion beam is shown in Fig. 9. Maximum glow peak intensity has been considered for fading analysis. Sample was stored for a period of seven weeks for fading analysis. During fading analysis no precautions was taken to protect the material from light or moisture. There is about 9.5% fading of TL intensity in the earlier two weeks. The next two weeks gives only 7% loss of TL signal. The total fading was 20% of the initial intensity for 155 °C TL glow peak over the complete duration of seven weeks. The huge amount of TL signal was faded due to the position of prominent TL glow peak which is around 155 °C. More study will be carried out in the future work to overcome the high fading property of the present material.

### Reproducibility

**Figure 10:**
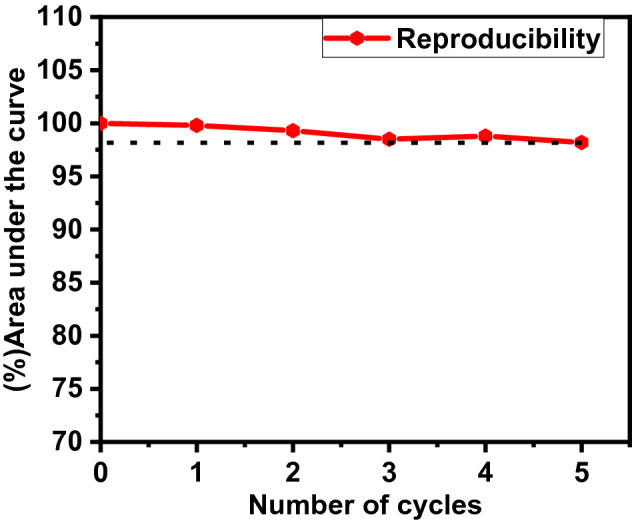
TL reproducibility curve of β-Ca_2_P_2_O_7_:(0.001)Dy nanocrystalline phosphor exposed to 1 × 10^11^ ions/cm^2^ of 75 meV C^6+^ ion beam over five cycles of annealing, irradiation and readout.

The reproducibility measurement of any material is an important parameter for the application propose. An ideal TLD material does not change its sensitivity and glow curve nature after several cycle of annealing, irradiation, and TL readout. 5 mg sample of β-Ca_2_P_2_O_7_:(0.001)Dy was used for five cycles of annealing, irradiation and TL readout. The observed percentage sensitivity of β-Ca_2_P_2_O_7_:(0.001)Dy phosphor after each cycle is shown in Fig. 10. The total loss of TL sensitivity is very less (only 2%) after five cycles of annealing, irradiation and readout. Hence, β-Ca_2_P_2_O_7_:Dy phosphor can be reused in radiation affected areas to measure the amount of radiation absorbed.

### Monte Carlo SRIM 2013 simulation

The influence of C^6+^ ion beam impact on the β-Ca_2_P_2_O_7_:Dy samples can be done by SRIM/ TRIM program by considering an incidence of one hundred thousand ions on a 1000 μm thick target of the present nanophosphor^35^. Vacancy distribution profile, Loss of energy, penetration depth, target ionization and other ion beam impact parameters were determined by considering the pellets of density 1.426 gm/cm^3^. The input data associated with ion beam parameter and target material was kept constant all over the simulations.

#### Energy loss, ion range and dose absorbed

**Figure 11:**
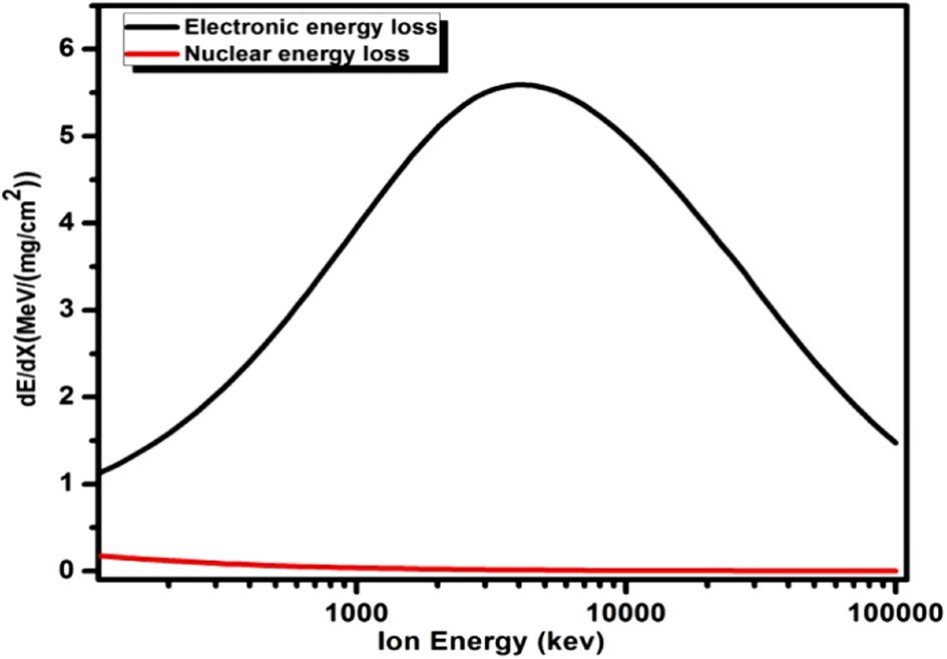
The electronic and nuclear energy loss calculated by SRIM-2013.

Interaction of HCP beams with targeted material causes to lose its energy via ionization or excitation of electrons and nuclear collision process known as electronic energy loss (S_e_) and nuclear energy loss (S_n_), respectively. Interaction type of ion beam with target material highly influence the TL characteristics^36^. Figure 11 displays the change in energy loss via electronic and nuclear stopping power of the β-Ca_2_P_2_O_7_:Dy phosphor for C^6+^ ion beam with energy ranging from 10 keV to 100 meV. Nuclear stopping power is negligible in comparison to electronic stopping power over the broad range of beam energy. Thus, electronic energy loss inside the β-Ca_2_P_2_O_7_:Dy phosphor appears to be responsible for the creation of color centers, electrons, holes, and exciton. On irradiating materials with HCPs produces deep tracks and creates additional deep traps in the materials. Hereafter, reorganization of luminescent or trapping centers modifies the TL characteristics to a large extent and can change TL properties of the thermoluminescent materials. LET is the total amount of energy absorbed by matter per unit length when charged ion travelled through it. The calculated value of LET was 1.837 meV/(mg/cm^2^) for C^6+^ ions of 75 meV energy. The range of 75 meV C^6+^ ion beam inside the phosphor is calculated to be 185.58 μm. Straggling, skewness, and kurtosis parameters are used to characterize the ion range profile. Theoretically obtained value of these set of parameters was calculated to be 1.5 μm, − 31.05 and 20.39, respectively. The obtained straggle value shows a lesser deviation in the ion range profile, while negative skewness value indicates that the distribution is skewed towards the surface. The distribution profile of ions will have flat broad tails for the kurtosis value greater than three.

Total delivered dose (D) after ion beam irradiation can be written by the equation^37^

1 $$ D = 1.602 \times 10^{ - 10} \times \frac{1}{\rho }\left( {\frac{dE}{{dx}}} \right) \times n $$

where D is in Gy, ion fluence (n) is in ion/cm^2^, density of the irradiated material (ρ) is in g/cm^3^, and LET (dE/dx) value is in MeVcm^2^/gm. The calculated values of equivalent dose with different ion fluences are summarized in Table 1.

**Table 1:** Dose absorbed by β-Ca_2_P_2_O_7_:Dy material at various fluences.

Ion beam	Fluence $\left( n \right)$	Dose $\left( D \right)$ kGy
C^6+^ ion beam 75 meV	2 × 10^10^	04.13
	5 × 10^10^	10.30
	1 × 10^11^	20.60
	5 × 10^11^	103.0
	1 × 10^12^	206.0

#### Target ionization and vacancy distribution profile

**Figure 12:**
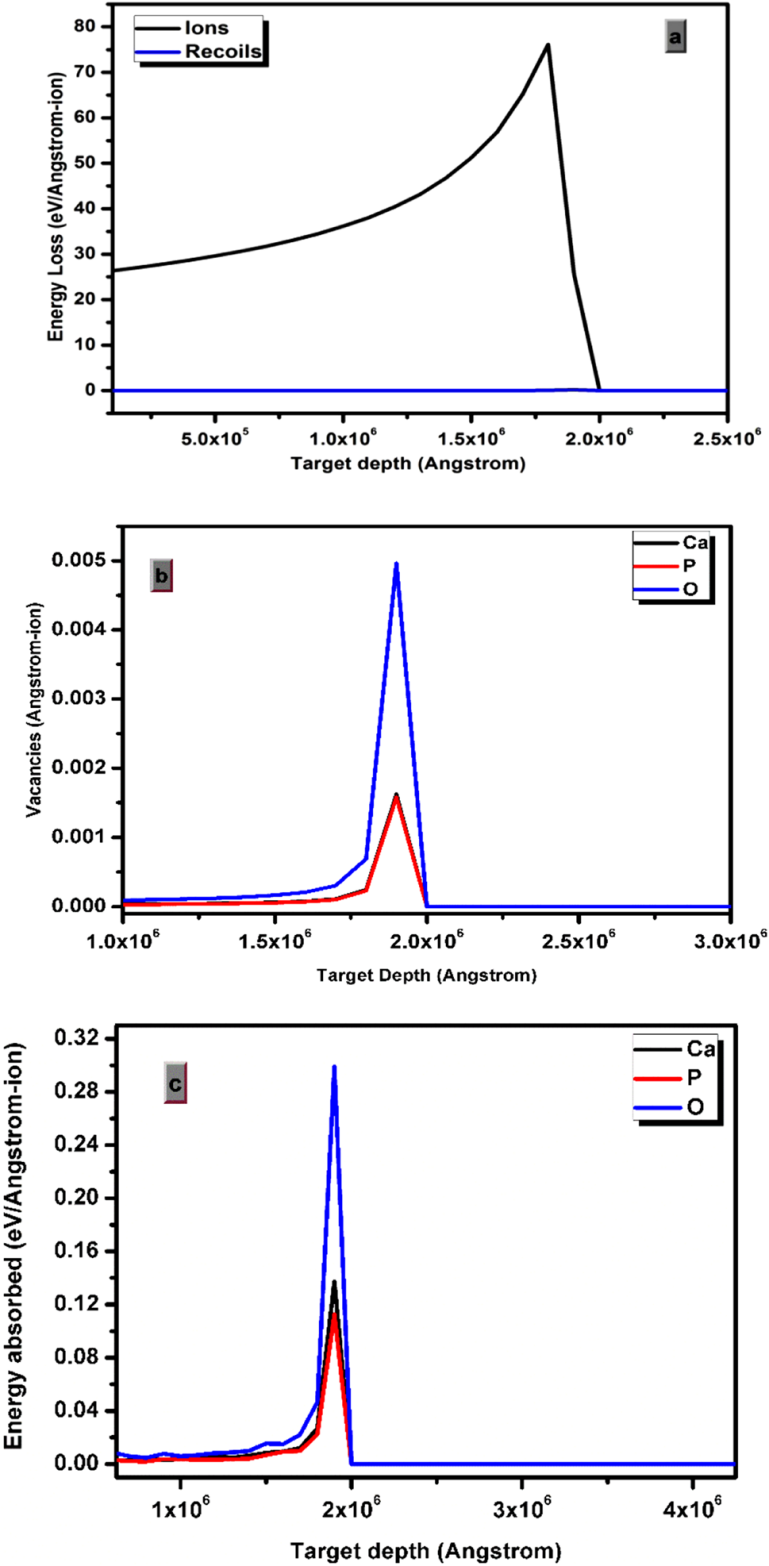
Simulated SRIM graphs of (**a**) target ionization (**b**) vacancy distribution in the target and (**c**) energy absorbed by the target atom as a function of target depth for the 75 meV C^6+^ ion beam.

Figure 12a displays the ionization profile behavior of target material. The energy loss of ions to the target electrons is small at the surface and increases exponentially with target depth. The energy loss of ions abruptly decreases to a minimum value after reaching a particular depth where it deposits its maximum energy. A non-uniform energy deposition nature of ions can be clearly observed through energy loss profile. Blue curve indicates the energy loss in the target material produced by recoil atoms. The energy loss has maximum peak value at 180 µm target depth. Recoil atoms has insignificant contribution to the energy loss in comparison to ionization produced by carbon ions. Vacancies are created inside β-Ca_2_P_2_O_7_:Dy phosphors when recoil atom changes position from its regular lattice site. Figure 12b shows the vacancy distribution profile of Ca, P, and O atoms in ion irradiated β-Ca_2_P_2_O_7_:Dy phosphor. Maximum vacancies are created at a depth of 190 µm for all Ca, P, and O ions. The number of oxygen vacancies is found to be created more as compared to other cation vacancies. Calcium and phosphorus vacancies are second highest in number. The oxygen vacancies will act as electron trapping centers while cation vacancies will act as hole trapping centers. This electron and hole trapping centres are accountable for the TL emission. However, all vacancies are not stable at normal room temperature due to recombination/trapping process. Oxygen vacancies are the most dominated due to its high absorption of ion energy during ion beam irradiation as shown in Fig. 12c. Electron trapping can be done by oxygen ion vacancies and can have a major role towards the TL process.

### Glow curve analysis

The analysis of trapping parameters is quite easy for a TL glow curve having only one single TL peak. Generally, the TL glow curve of most of the TLD phosphor is found to be comprises of more than one TL peak which may overlapped with each other. In such situation the calculation of trapping parameters become complex in nature. Thus, we have to separate each and every TL glow peak to calculate the trapping parameters of each peak. This can be done by using several kinetics equations. In our study we have used GCD functions developed by Kitis et al*.* for different kinetic orders^38^. To use these equations first of all we have to make a rough estimation of some initial parameters to generate theoretical glow curve and separated from experimental glow curve. These initial parameters are nothing but the estimation of possible number of TL glow curves and their maximum peak intensity, order of kinetics and maximum peak temperature of each peak. These values were varied until a good fit between theoretical and experimental glow curve was observed. The FOM value was used to determine the best fitting of theoretical glow curve with experimental one. The calculated value of FOM using Eq. (3) is found to be 0.023%. The recorded glow curve was investigated for the separation of each and every peak by considering first order, general order and second order kinetics. The GCD function used for the separation of each TL glow peak is given below.

For general order,

2 $$ I = I_{m} b^{{\left( {\frac{b}{b - 1}} \right)}} \exp \left( {\frac{E}{kT}\frac{{T - T_{m} }}{{T_{m} }}} \right) \times \left[ {\left( {b - 1} \right)\left( {1 - \frac{2kT}{E}} \right)\frac{{T^{2} }}{{T_{m}^{2} }}\exp \left( {\frac{E}{kT}\frac{{T - T_{m} }}{{T_{m} }}} \right) + 1 + \left( {b - 1} \right)\frac{{2kT_{m} }}{E}} \right]^{{ - \left( {\frac{b}{b - 1}} \right)}} $$

3 $$ FOM = \frac{{\mathop \sum \nolimits_{i} Y_{i} - Y_{{{\text{exp}}}} }}{{\mathop \sum \nolimits_{i} Y_{i} }} $$

where, I(T) is the TL intensity at a particular temperature T (K), I_m_ is the maximum peak intensity, k is the Boltzmann constant, and E is the activation energy (eV).

Some of the initial input parameters such as order of kinetics, and activation energy needed for the isolation of each peak was determined by the Chen’s peak shape method^39^. This method is useful in finding the order of kinetics of each individual peak by using only the shape parameters like δ and ω. Here δ is T_2_ − T_m_ (half width of TL glow peak on higher temperature side) and ω is T_2_ − T_1_ (total half width of each TL glow peak) can be calculated by using only the glow peak shape of each TL glow curve. Symmetry factor μ_g_ can be determined by using the ratio between δ and ω. The obtained value of μ_g_ decides the value of b according to relation reported by Chen et al.^39^ the value of trapping parameters calculated by Chen’s peak shape method is listed in Table 2. The various equations used for the calculation trapping parameters are shown below.

**Table 2:** Trapping parameters by using Chen’s peak shape method.

Sample	Peak no. (°C)	Geometrical form factor (µ_g_)	Activation energy (eV)	Frequency factor (s^−1^)
β-Ca_2_P_2_O_7_:(0.001)Dy	1 (152)	0.52	1.26	4.60 × 10^14^
	2 (206)	0.48	1.29	1.27 × 10^13^
	3 (240)	0.52	1.31	1.87 × 10^12^
	4 (296)	0.51	1.34	1.90 × 10^11^

The symmetry factor can be written as

4 $$ \mu_{g} = \frac{{T_{2} - T_{m} }}{{T_{2} - T_{1} }} $$

The obtained value of symmetry factor was substituted in Eq. (5) to calculate activation energy of TL glow peaks.

5 $$E_{\alpha } = c\left( {\frac{{kT_{m}^{2} }}{\alpha }} \right) - b_{{}} \left( {2kT_{m} } \right)$$

where, α stands for *τ,* *δ* *or* *ω.* The values of *c*_*γ*_ and *b*_*γ*_ are summarized as below

6 $$ c_{\tau } = 1.510 + 3.0\left( {\mu_{g} - 0.42} \right),\;b_{\tau } = 1.58 + 4.2\left( {\mu_{g} - 0.42 } \right) $$

7 $$ c_{\delta } = 0.976 + 7.3\left( {\mu_{g} - 0.42 } \right),\;b_{\delta } = 0 $$

8 $$ c_{\omega } = 2.52 + 10.2\left( {\mu_{g} - 0.42} \right),\;b_{\delta } = 1 $$

As the value of activation energy was calculated it was substituted to Eq. (9) to obtain the value of frequency factor.

9 $$ s = \frac{E}{{kT_{m}^{2} }}\exp \left( {\frac{E}{{kT_{m} }}} \right)\left[ {1 + \left( {b - 1} \right)\Delta_{m} } \right] $$

$\Delta_{m} = \frac{{2kT_{m} }}{E}$, k is Boltzman’s constant and β is linear heating rate.

**Figure 13:**
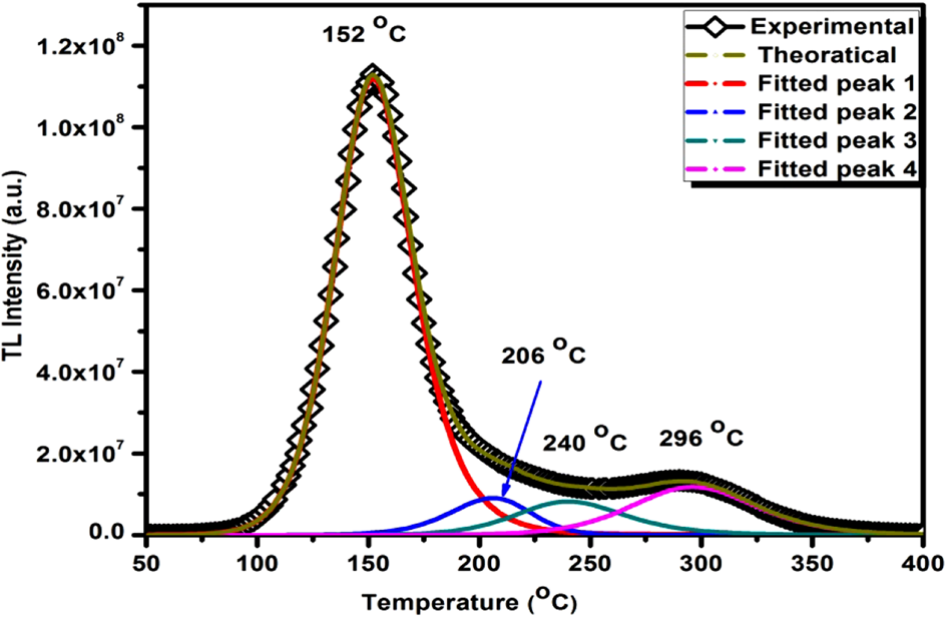
Comparison between the experimental and theoretically fitted TL glow curve of the β-Ca_2_P_2_O_7_:(0.001)Dy phosphor exposed to 2 × 10^10^ ions/cm.

Once the value of trapping parameters was calculated using above mentioned equations, it was used to generate the theoretical glow curve by using GCD functions. The best value of trapping parameters for which the theoretical and experimental data matched very well was set fixed. The trapping parameters values obtained by GCD functions are listed in Table 3. The experimental and theoretically fitted TL glow peak is shown in Fig. 13.

**Table 3:** Trapping parameters by using GCD functions.

Sample	Peak no	Temperature T_m_ (°C)	Order of kinetics	Activation energy (eV)	Frequency factor (s^−1^)
β-Ca2P2O7:(0.001)Dy	1	152	2	1.24	2.14 × 10^14^
	2	206	1.4	1.28	9.74 × 10^12^
	3	240	2	1.30	1.81 × 10^12^
	4	296	1.8	1.33	1.23 × 10^11^

### Thermoluminescence mechanism

**Figure 14:**
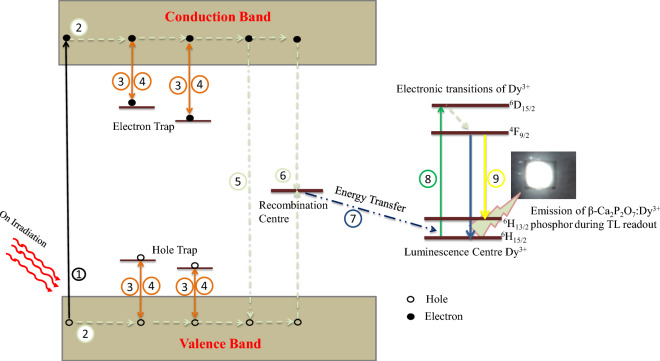
The energy bands model for the TL process occurring in β-Ca_2_P_2_O_7_:Dy^3+^ phosphors.

Figure 14 depicted an illustration of the processes occur during ion beam irradiation and subsequent measurement of TL signal through energy band diagram of β-Ca_2_P_2_O_7_. On irradiation a number of free electron (e^−^) and holes (h^+^) are produced by absorbing the energy of C^6+^ ion beam by β-Ca_2_P_2_O_7_ (process 1). The free charge carriers are travel through the crystalline structure and get trapped at their respective defect centers (process 2&3). The defect centers formed in β-Ca_2_P_2_O_7_ may be due the several reasons in which the incorporation of trivalent dopant ion at divalent calcium ion is crucial one. The incorporation of Dy^3+^ ion in the host matrix creates point defects of calcium vacancies ($V^{\prime\prime}_{Ca}$) (a negative defect) and dysprosium substitution ($RE^{\prime}_{Ca}$) (two positive defect) to maintain the electrical neutrality of the material via charge compensation. In β-Ca_2_P_2_O_7_, three Ca^2+^ atoms will be replaced by two Dy^3+^ atoms to maintain the charge neutrality of the system. These $RE^{\prime}_{Ca}$ and $V^{\prime\prime}_{Ca}$ defects are act as trapping centers for electrons and holes, respectively. Some of the charge carriers get recombines with their counterpart during the irradiation stage via radiative and non-radiative way which results radio-luminescence phenomenon. However, the TL phenomenon was occurred when the external thermal energy was given to the trapped electrons and holes to escape from their respective traps and recombine radiatively or non-radiatively at valence band or at recombination center (process 5&6). The released charge carriers sometime get re-trapped (process 4) or recombine non-radiatively. The recombination energy was transferred to the activator ion (process 7) to make it in excited state (process 8) such that the de-excitation process gives the luminescence (process 9) which was recorded as a TL glow curve during TL measurements. Dy^3+^ is acting as luminescence center in β-Ca_2_P_2_O_7_ host during TL measurements because we have only observed white emission from the host and shown in Fig. 14. The white emission is the characteristics of Dy^3+^ ion and it is already mentioned in the PL measurement section. According to the previous study, the Dy^3+^ and P_2_O_7_^4−^ ion can also act as trapping centers for electrons and holes^15^. The TL peak observed at higher temperature side is due to the oxygen vacancies created by highly energetic C^6+^ ion beam during irradiation. The conformation of oxygen vacancies is already proved by the SRIM calculations in Sect. 3.9. The oxygen vacancies will trap electron and act as electron trapping centers.

## Conclusion

The series of nonocrystalline β-Ca_2_P_2_O_7_:Dy^3+^ (x = 0.0005, 0.001, 0.003 and 0.005) phosphors has been prepared successfully using wet chemical method. The XRD result shows the prominent diffraction peaks of tetragonal structure of β-Ca_2_P_2_O_7_. FTIR spectra of β-Ca_2_P_2_O_7_:(0.001Dy) phosphor confirms the absence of water molecule in the as synthesized sample. The actual crystallite size of β-Ca_2_P_2_O_7_:Dy nanophosphor was determined from the TEM analysis. TEM image shows highly agglomerated non-uniform nanoparticles having average grain size of less than 30 nm which is comparable with the crystallite size predicted by SEM data. The presence of emission peaks at 482 nm and 576 nm during PL measurement and absorption peaks at 454 nm, 388 nm, 366 nm and 352 nm in the excitation spectra is the confirmation of dopant ion in the host matrix of β-Ca_2_P_2_O_7_ phosphor. The TL study of the phosphors has been carried out under 75 meV C^6+^ ion beam exposure. The TL glow curve has four glow peaks, which may be due to the formation of more number of trap levels after highly ionizing heavy ion beam irradiation. The de-convolution of complex glow curve was carried out using GCD functions and trapping parameters were analyzed using both GCD and Chen’s peak shape methods. A good matching between the trapping parameter values calculated via both methods is observed. Moreover, the 155 °C TL glow peak exhibits a wide linear TL response in the range 1 × 10^10^–1 × 10^11^ ions/cm^2^. The linear and sub-linear behavior of TL response curve was discussed in the frame work of TIM. The sub-linear effect was found to be occurring due to the overlapping of ion tracks in the material at higher doses. The main energy loss, absorbed doses, ion range and depth for maximum vacancy formation were calculated using TRIM code based on the Monte Carlo simulation. These results showed that it is quite possible to use β-Ca_2_P_2_O_7_:Dy nanoparticles as a dosimeter for C ion beam due to its wide linear response along with the high stability of TL glow curve.
